# No Significant Differences in Antidepressant Remission and Response Rates Between Anxious and Nonanxious Depression: A Multicenter Study in Taiwan

**DOI:** 10.1155/da/3138685

**Published:** 2026-11-10

**Authors:** Wei-Yang Lee, Hsiao-Hui Tsou, Si-Yu Chu, Ya-Ting Hsu, Chia-Yih Liu, Chin-Fu Hsiao, Huang-Li Lin

**Affiliations:** ^1^ Department of Psychiatry Chang Gung Memorial Hospital Linkou Taoyuan Taiwan cgmh.org.tw; ^2^ Institute of Population Health Sciences National Health Research Institutes Zhunan Miaoli Taiwan nhri.org.tw; ^3^ Graduate Institute of Biostatistics College of Public Health China Medical University Taichung Taiwan cmu.edu.cn; ^4^ Department of Psychiatry Taipei City Psychiatric Center Taipei City Hospital Taipei Taiwan tch.gov.taipei; ^5^ Department of Psychiatry New Taipei City Tucheng Hospital New Taipei Taiwan; ^6^ College of Medicine Chang Gung University Taoyuan Taiwan cgu.edu.tw

**Keywords:** anxious depression, Beck Depression Inventory-II, escitalopram and paroxetine, Hamilton Rating Scale for Depression, nonanxious depression

## Abstract

**Background:**

Anxious depression, defined by coexisting anxiety and depressive symptoms, has been associated with poorer treatment outcomes, though findings remain inconsistent. This study examined whether anxious depression influences antidepressant efficacy in an Asian population.

**Methods:**

This multicenter, single‐blind study across five hospitals was conducted in Taiwan from May 2006 to December 2009. Patients aged 18 and older with major depressive disorder (MDD) were diagnosed and treated with either escitalopram or paroxetine. The Hamilton Rating Scale for Depression (HAM‐D) and the Beck Depression Inventory‐II (BDI‐II) assessed outcomes. Anxious depression was defined as a HAM‐D anxiety factor score ≥ 7. We assessed treatment efficacy by remission and response rates, as well as changes in depression scores.

**Results:**

The study included 302 patients, of whom 193 were classified as having anxious depression and 109 as having nonanxious depression. No significant differences were observed in treatment dosage or duration between the anxious and nonanxious depression groups. HAM‐D‐defined remission rates were 40.4% and 43.1%, respectively (adjusted OR = 0.9, 95% CI = 0.6–1.5, and *p* = 0.651), and response rates were 62.2% and 56.9% (adjusted OR = 1.2, 95% CI = 0.7–1.9, and *p* = 0.517). The anxious depression group showed a greater reduction in HAM‐D scores (−13.0 vs. −10.4; adjusted mean difference = −1.9, 95% CI = −3.5 to −0.4, and *p* = 0.014), whereas the percentage change did not differ significantly between groups (adjusted *p* = 0.529). BDI‐II outcomes likewise showed no significant between‐group differences. Time to remission (log‐rank *p* = 0.599) and response (log‐rank *p* = 0.264) also did not differ significantly.

**Conclusions:**

In this Taiwanese multicenter outpatient cohort, anxious depression was not significantly associated with lower remission or response rates during 8 weeks of treatment with escitalopram or paroxetine. These findings contribute to the limited evidence on antidepressant outcomes among Asian outpatients with anxious depression. However, the absence of statistically significant differences should not be interpreted as evidence of equivalence, and further prospective studies with larger and better‐balanced samples are needed to confirm these findings.

**Trial Registration:** ClinicalTrials.gov identifier: NCT00384020

## 1. Introduction

Depression and anxiety are associated with substantial physical problems and high mortality rates. The coexistence of depression and anxiety considerably raises medical utilization and is associated with increased chronicity, slower recovery, higher recurrence rates, and more significant psychosocial disability [1]. These individuals are also more likely to have melancholic features, be older, experience more severe depression, have a higher risk of suicide, endure more pain, have a lower quality of life, and face more severe functional impairment [2].

Regarding the prevalence of anxious depression, a global survey found that 45.7% of individuals with lifetime major depressive disorder (MDD) also had a history of one or more anxiety disorders [3]. Anxious depression was reported in 53.2% of patients with depressive disorder [4]. Another European multicenter study identified that 286 participants (21.2%) had comorbid anxiety disorders: 10.8% had generalized anxiety disorder (GAD), 8.3% had panic disorder, 8.1% had agoraphobia, and 3.3% had social phobia. Notably, comorbid GAD is associated with high symptom severity and poor treatment response [5].

Asian populations show higher prevalence rates. In China, 64.5% of outpatients with treatment‐resistant depression (TRD) had anxious depression [6]. A study in Taiwan estimated that 81% of 174 inpatients with MDD had anxious depression [2]. In our previous research, 64.16% of participants with depression were classified as having anxious depression [7].

Past research indicates that anxious depression is more difficult to treat. Patients with anxious depression are much less likely to respond to treatment, sustain response or remission, and take a longer time to recover compared to those with nonanxious MDD [8–10]. These patients frequently report a more significant burden of side effects than those with nonanxious depression [9]. The presence of comorbid anxiety disorders with depression predicts lower persistence in MDD treatment [11]. Previous research found that anxious depression was linked to a poorer response to antidepressant therapy, lower remission rates, and longer recovery times, and that patients may experience poorer treatment responses following antidepressant therapy [4]. Furthermore, anxious depression is linked to significantly higher suicidal ideation and is consistently associated with increased mortality [3, 12]. However, in contrast to the previous discussion, a study suggests that the presence of anxiety as a comorbid condition does not further increase the risk of death in those with depressive disorder [13]. Other research indicates that anxiety may not elevate mortality rates, even among individuals aged over 60. A small protective effect was noted regarding the occurrence of any anxiety disorder when adjusted for lifestyle characteristics, multimorbidity, and frailty [14]. The reasons may be that symptoms associated with anxiety, such as digestive discomfort and decreased appetite, could potentially contribute to lower mortality, possibly due to the increased use of healthcare services [13].

To the best of our knowledge, most previous studies have suggested that anxious depression is associated with greater symptom severity and poorer treatment outcomes than nonanxious depression. However, anxiety does not appear to be a uniformly negative prognostic factor across all studies. Moreover, evidence comparing antidepressant treatment outcomes between anxious and nonanxious depression remains limited in Asian populations, particularly in Taiwan. Given the reported cross‐cultural differences in the prevalence and clinical presentation of anxious depression, we hypothesized that anxious depression would be associated with poorer treatment outcomes than nonanxious depression in Taiwanese outpatients. However, the magnitude of this association might differ from that reported in Western populations. Therefore, our investigation aims to examine a multicenter study in Taiwan to explore in Asian populations: (1) whether anxious depression is also associated with poorer prognosis and greater severity of depression and (2) whether anxious depression impacts medication efficacy more than nonanxious depression.

## 2. Methods

### 2.1. Study Participants

From May 2006 to December 2009, we recruited patients from the outpatient psychiatric clinics of five hospitals in and around Taipei, Taiwan: Chang Gung Memorial Hospital, Mackay Memorial Hospital, Far Eastern Memorial Hospital, TMU‐Wan Fang Hospital, and the Songde branch of Taipei City Hospital. Based on their parents’ documented backgrounds, these patients were identified as Han Chinese. Inclusion criteria were (1) over 18 years old, (2) currently experiencing a depressive episode confirmed during an interview, and (3) scoring more than 14 on the 21‐item Hamilton Rating Scale for Depression (HAM‐D). All participants were interviewed by trained research nurses using the Structured Clinical Interview for DSM‐IV‐TR Axis‐I Disorders (SCID), and MDD was diagnosed based on DSM‐IV‐TR criteria [15]. Individuals with comorbid conditions such as schizophrenia, bipolar disorder, schizoaffective disorder, substance dependence, dementia, or any significant medical condition were excluded from the study. Additionally, those previously treated with escitalopram or paroxetine were also excluded. All participants were thoroughly informed and provided written consent, obtained in accordance with the guidelines of the Declaration of Helsinki, before enrolling in the study.

### 2.2. The Treatment Protocol

Before starting the protocol, patients had to complete a 7‐day washout period for previous antidepressant treatments (12 days for fluoxetine). Clinicians were not blinded to treatment allocation, which was determined by clinical judgment. Participants were unaware of whether they received escitalopram or paroxetine during the treatment period. In the escitalopram group, patients received a fixed daily dose of 10 mg for the first 4 weeks, followed by flexible dosing of 10–30 mg/day, depending on their clinical response over 8 weeks. In the paroxetine group, patients received a fixed daily dose of 20 mg for the first 4 weeks, followed by flexible dosing of 20–40 mg/day for the next 4 weeks. No other psychotropic drugs were permitted, except for 10 mg of zolpidem per night for insomnia (as needed, up to four nights per week) and 1–2 mg of lorazepam per day for anxiety as needed. The study was approved by the Institutional Review Board of the National Health Research Institutes in Taiwan (NSC 95‐2314‐B‐400‐001). Additional details are available at the provided link: https://classic.clinicaltrials.gov/ct2/show/NCT00384020. The study was also registered under the Chang Gung Memorial Hospital Research Program (CMRPG CORPG3L0021). This study was a secondary analysis of data collected in the original clinical trial, and the analyses of anxious depression and treatment outcomes were not prespecified or preregistered in the original study protocol.

### 2.3. Measurement

Raters were blinded to the patients’ conditions and the prescribed medications. The patients who completed the measurements at weeks 4, 6, or 8 were included in data analysis.

#### 2.3.1. Anxiety and Depression Scales

The 21‐item HAM‐D was used to assess depression, conducted by experienced healthcare providers and widely used in clinical trials [16]. The scale’s items are grouped into several domains, including core depressive symptoms, sleep patterns, daily activities, various aspects of anxiety (both psychic and somatic), and delusions. For this study, anxious depression was defined as having a score of ≥7 on the HAM‐D anxiety factor. Items contributing to the anxiety/somatization factor on the HAM‐D include item 10 (anxiety—psychic), item 11 (anxiety—somatic), item 12 (somatic symptoms—gastrointestinal), item 13 (somatic symptoms—general), item 15 (hypochondriasis), and item 17 (insight). Scores on this scale ranged from 0 to 18.

In addition to clinician‐administered assessments, the Beck Depression Inventory‐II (BDI‐II) was used as a self‐report depression scale [17]. This scale consists of 21 items, each rated from 0 to 3. The Chinese Behavioral Science Corporation translated and validated the BDI‐II into Mandarin Chinese [18].

### 2.4. Statistical Analysis

All statistical analyses were performed using the Statistical Analysis System (SAS) Version 9.1 software (SAS Institute, Cary, North Carolina, USA). The treatment characteristics of patients, categorized by the presence of anxious depression or nonanxious depression, were analyzed using the Chi‐squared test or Fisher’s exact test, depending on the distribution of the variables.

We defined the response as a ≥50% reduction from baseline in the HAM‐D score at the endpoint. Remission was defined as achieving a HAM‐D score of ≤7 or a BDI‐II score of ≤8 at the endpoint. We analyzed the response and remission rates using logistic regression models to estimate the odds of achieving these outcomes. HAM‐D and BDI‐II exit scores were evaluated using general linear models to assess the mean differences between anxious depression and nonanxious depression groups. To control for potential confounding variables, the analyses were adjusted for hospital site and baseline severity of depression, measured on both the HAM‐D (excluding anxiety‐related factors) and the BDI‐II.

Furthermore, the association between the presence of anxiety and/or anxious depression and the likelihood of achieving remission, as assessed by either the HAM‐D or BDI‐II, was evaluated using logistic regression models. The time to remission and response was analyzed using product‐limit survival estimates (Kaplan–Meier method) to compare differences between the anxious and nonanxious depression groups [19]. We used log‐rank tests to assess the statistical significance of differences in survival distributions between groups. We set the significance level for all statistical tests at *α* = 0.05 (two‐sided).

## 3. Results

### 3.1. Participant Characteristics

In this study, we included 302 of the 399 eligible participants in the evaluable sample. All included participants had a baseline HAM‐D score of 14 or higher and had available follow‐up data through at least Week 4 during the 8‐week medication trial. We excluded the remaining 97 participants because of insufficient follow‐up or missing outcome data. For participants who missed the Week 6 or Week 8 assessments, the last available observation was carried forward (LOCF) for outcome analysis. Figure 1 presents the overall participant flow, including eligibility, exclusions, attrition, and the final analyzable sample. In the analysis, 193 (63.9%) were classified as having anxious depression and 109 (36.1%) as having nonanxious depression. Baseline sociodemographic and clinical characteristics differed between the two groups. Compared with patients with nonanxious depression, those with anxious depression tended to be older, had fewer years of education, were more likely to be married and to have children, and had a later age at first depressive episode. They also exhibited greater baseline clinical severity, including higher HAM‐D and CGI‐S scores, and a higher prevalence of panic disorder without agoraphobia. We reported these baseline characteristics in greater detail in our previous study [20]. Figure 2 shows the distribution of HAM‐D anxiety/somatization factor scores for the analyzable sample.

**Figure 1:**
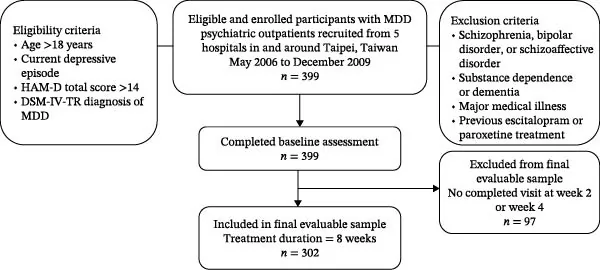
Participants flow diagram. HAM‐D, Hamilton Rating Scale for Depression; MDD, major depressive disorder.

**Figure 2:**
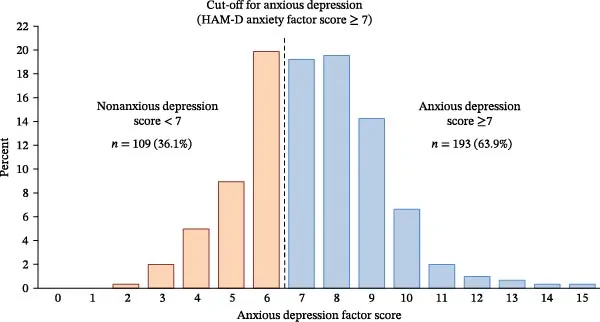
Distribution of HAM‐D anxiety/somatization (anxious depression) factor scores (*N* = 302). The anxiety/somatization factor includes six items from the 21‐item HAM‐D, including anxiety (psychic), anxiety (somatic), somatic symptoms (gastrointestinal), somatic symptoms (general), hypochondriasis, and insight.

Table 1 shows that there is no significant difference in medication dosage between anxious and nonanxious depression groups, whether escitalopram or paroxetine is used for treatment. Treatment duration also did not differ significantly between the two groups. These findings suggest that both medications and treatment duration were comparable for both types of depression.

**Table 1:** Treatment characteristics of patients by presence of anxious depression.

Dose and treatment	Nonanxious depression (*N* = 109)		Anxious depression (*N* = 193)		Total (*N* = 302)		*p* ^a^
	*n*	%	*n*	%	*n*	%	
Maximum escitalopram dosage (mg)	*n* = 36	—	*n* = 78	—	*n* = 114	—	0.5924
<10	1	2.8	7	9.0	8	7.0	
10–20	32	88.9	64	82.1	96	84.2	
≥20	3	8.3	7	9.0	10	8.8	
Escitalopram dosage at study exit (mg)	*n* = 36	—	*n* = 78	—	*n* = 114	—	0.1328
<10	2	5.6	15	19.2	17	14.9	
10–20	32	88.9	57	73.1	89	78.1	
≥20	2	5.6	6	7.7	8	7.0	
Maximum paroxetine dosage (mg)	*n* = 73	—	*n* = 115	—	*n* = 188	—	0.7826
<20	7	9.6	9	7.8	16	8.5	
20–40	58	79.5	96	83.5	154	81.9	
≥40	8	11.0	10	8.7	18	9.6	
Paroxetine dosage at study exit (mg)	*n* = 73	—	*n* = 115	—	*n* = 188	—	0.2122
<20	17	23.3	17	14.8	34	18.1	
20–40	48	65.8	89	77.4	137	72.9	
≥40	8	11.0	9	7.8	17	9.0	
Time in treatment (weeks)	—	—	—	—	—	—	0.3843
4–8	8	7.3	20	10.4	28	9.3	
= 8	101	92.7	173	89.6	274	90.7	

^a^Chi‐square test or Fisher’s exact test.

### 3.2. Remission and Response in Patients With Anxious and Nonanxious Depression

As shown in Table 2, HAM‐D‐defined remission was achieved by 40.4% of patients with anxious depression and 43.1% of those with nonanxious depression, with no statistically significant between‐group difference after adjustment for hospital site and baseline depression severity (adjusted OR = 0.9, 95% CI = 0.6–1.5, and *p* = 0.6508). Similarly, among participants with available BDI‐II data, 30.2% of the anxious depression group and 39.8% of the nonanxious depression group achieved BDI‐II‐defined remission (adjusted OR = 0.7, 95% CI = 0.4–1.1, and *p* = 0.1357). HAM‐D‐defined response rates were also not significantly different between the anxious and nonanxious depression groups (62.2% vs. 56.9%, respectively; adjusted OR = 1.2, 95% CI = 0.7–1.9, *p* = 0.5165). Continuous symptom outcomes showed a similar pattern. The mean HAM‐D exit score was 10.5 ± 6.8 in the anxious depression group and 9.3 ± 5.7 in the nonanxious depression group, with no significant adjusted between‐group difference (adjusted mean difference = 1.1, 95% CI = −0.4 to 2.6, and *p* = 0.1398). However, the anxious depression group showed a greater reduction in HAM‐D scores from baseline (−13.0 ± 7.4 vs. −10.4 ± 6.5; adjusted mean difference = −1.9, 95% CI = −3.5 to −0.4, and *p* = 0.0137). In contrast, the percentage reduction in HAM‐D scores did not differ significantly between groups (−54.8 ± 28.1 vs. −51.6 ± 30.4; adjusted mean difference = −2.2, 95% CI = −9.0 to 4.6, and *p* = 0.5291). For BDI‐II, we observed no significant between‐group differences in the exit score, absolute change, or percentage change. The mean BDI‐II exit score was 17.0 ± 13.3 in the anxious depression group and 14.9 ± 12.0 in the nonanxious depression group (adjusted mean difference = 1.1, 95% CI = −1.6 to 3.8, and *p* = 0.4066). The mean absolute change in BDI‐II score was −16.1 ± 12.0 and −16.2 ± 13.0, respectively (adjusted mean difference = 1.1, 95% CI = −1.6 to 3.8, and *p* = 0.4066), while the percentage change was −49.6 ± 32.5 and −50.3 ± 35.7, respectively (adjusted mean difference = 1.2, 95% CI = −6.8 to 9.2, and *p* = 0.7724).

**Table 2:** Remission and response in patients, by presence of anxious depression.

Outcome	Anxious depression							OR (95% CI)	*p* ^a^	OR (95% CI)	Adjusted *p* ^b^
	No (*N* = 109)		Yes (*N* = 193)		Total (*N* = 302)						
	*n*	%	*n*	%	*n*		%				
Remission (score ≤ 7 on 21‐item HAM‐D)								0.9 (0.6, 1.4)	0.6468	0.9 (0.6, 1.5)	0.6508
No	62	56.9	115	59.6	177		58.6				
Yes	47	43.1	78	40.4	125		41.4				
Remission (score ≤ 8 on BDI) (*n* = 297)								0.7 (0.4, 1.1)	0.0912	0.7 (0.4, 1.1)	0.1357
No	65	60.2	132	69.8	197		66.3				
Yes	43	39.8	57	30.2	100		33.7				
Response (≥50% reduction from baseline on 21‐item HAM‐D)								1.2 (0.8, 2.0)	0.3668	1.2 (0.7, 1.9)	0.5165
No	47	43.1	73	37.8	120		39.7				
Yes	62	56.9	120	62.2	182		60.3				
	Mean	SD	Mean	SD	Mean		SD	Mean difference (95% CI)	—	Mean difference (95% CI)	—
HAM‐D exit score	9.3	5.7	10.5	6.8	10.1		6.4	1.6 (−0.3, 2.8)	0.1035	1.1 (−0.4, 2.6)	0.1398
Change in score	−10.4	6.5	−13	7.4	−12.1		7.2	−2.6 (−4.3, −0.9)	**0.0025**	−1.9 (−3.5, −0.4)	**0.0137**
% change in score	−51.6	30.4	−54.8	28.1	−53.6		28.9	−3.2 (−10.0, 3.6)	0.3591	−2.2 (−9.0, 4.6)	0.5291
BDI exit score	14.9	12.0	17.0	13.3	16.2		12.8	2.0 (−1.0, 5.1)	0.1912	1.1 (−1.6, 3.8)	0.4066
Change in score	−16.2	13.0	−16.1	12.0	−16.1		12.4	0.1 (−2.8, 3.1)	0.9386	1.1 (−1.6, 3.8)	0.4066
% change in score	−50.3	35.7	−49.6	32.5	−49.9		33.6	0.7 (−7.3, 8.7)	0.8585	1.2 (−6.8, 9.2)	0.7724

*Note:* Bold values indicate statistical significance.

Abbreviations: BDI, Beck Depression Inventory; HAM‐D, Hamilton Rating Scale for Depression.

^a^Remission and response rates were analyzed using logistic regression models; HAM‐D exit scores and BDI exit score were analyzed using general linear models.

^b^After adjusting for hospital and baseline severity of depression on the Hamilton Depression Rating Scale without anxiety factors or on the Beck Depression Inventory, remission and response rates were analyzed using logistic regression models; HAM‐D exit scores and BDI exit score were analyzed using general linear models. The specific items contributing to the anxiety/somatization (anxious depression) factor scores in the HAM‐D included items 10 (anxiety—psychic), 11 (anxiety—somatic), 12 (somatic symptoms—gastrointestinal), 13 (somatic symptoms—general), 15 (hypochondriasis), and 17 (insight).

Table 3 examined the associations between four anxiety‐related variables and remission as defined by the HAM‐D and BDI‐II. None of the anxiety‐related variables was significantly associated with either remission outcome. For the presence of anxious depression, the OR was 0.90 (95% CI = 0.56–1.44) for HAM‐D‐defined remission and 0.65 (95% CI = 0.40–1.07) for BDI‐II‐defined remission. For the total HAM‐D anxiety/somatization factor score, the OR was 1.01 (95% CI = 0.90–1.14) for HAM‐D‐defined remission and 1.00 (95% CI = 0.88–1.12) for BDI‐II‐defined remission. For the total HAM‐D psychic and somatic anxiety score, the OR was 0.94 (95% CI = 0.80–1.09) for HAM‐D‐defined remission and 0.87 (95% CI = 0.74–1.02) for BDI‐II‐defined remission. For the presence of any SCID‐defined comorbid anxiety disorder, the OR was 0.82 (95% CI = 0.50–1.32) for HAM‐D‐defined remission and 0.72 (95% CI = 0.43–1.18) for BDI‐II‐defined remission. The *R* ^2^ and max‐rescaled *R* ^2^ values were low across all models, indicating that these anxiety‐related variables accounted for only a small proportion of the variation in remission outcomes.

**Table 3:** Association between presence of anxiety and/or anxious depression and treatment outcome in patients.

Anxiety variable	Remission per HAM‐D (score ≤ 7)					Remission per BDI (score ≤ 8)				
	Odds ratio	95% CI		*R* ^2^	Max‐rescaled *R* ^2^	Odds ratio	95% CI		*R* ^2^	Max‐rescaled *R* ^2^
Presence of anxious depression	0.90	0.56	1.44	0.0007	0.0009	0.65	0.40	1.07	0.0095	0.0132
Total score on HAM‐D anxiety/somatization factor^a^	1.01	0.90	1.14	0.0002	0.0002	1.00	0.88	1.12	0.0000	0.0000
Total score on HAM‐D psychic and somatic anxiety items^b^	0.94	0.80	1.09	0.0024	0.0032	0.87	0.74	1.02	0.0101	0.0140
Presence of any comorbid anxiety disorders^c^	0.82	0.50	1.32	0.0023	0.0031	0.72	0.43	1.18	0.0057	0.0079

Abbreviations: BDI, Beck Depression Inventory; HAM‐D, Hamilton Rating Scale for Depression.

^a^Total score on HAM‐D anxiety/somatization factor = anxiety (psychic) + anxiety (somatic) + somatic symptoms (gastrointestinal) + somatic symptoms (general) + hypochondriasis + insight.

^b^Total score on HAM‐D psychic and somatic anxiety items = anxiety (psychic) + anxiety (somatic) + somatic symptoms (gastrointestinal) + somatic symptoms (general).

^c^The presence of any comorbid anxiety disorder was defined as meeting one of the anxiety disorders of the Structured Clinical Interview for DSM (SCID).

Figures 3 and 4 illustrate the time to remission and time to response, respectively, comparing the anxious depression group to the nonanxious depression group. In Figure 3, the log‐rank test revealed no significant difference in the time to remission between the two groups (*p* = 0.5985). Similarly, Figure 4 shows that the time to response did not differ significantly between the groups (*p* = 0.2638). These results suggest that despite the presence of anxiety in depression, it does not significantly impact the duration to achieve remission or response to treatment.

**Figure 3:**
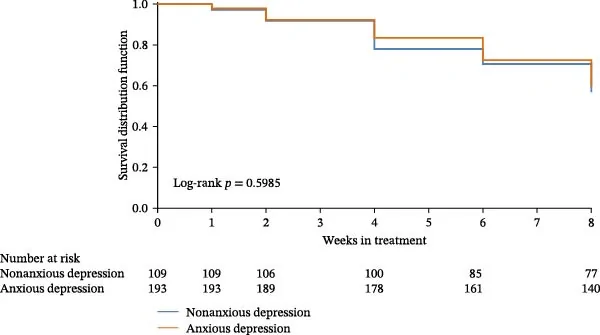
Time to remission, by anxious versus nonanxious depression. Log‐rank *p* = 0.5985.

**Figure 4:**
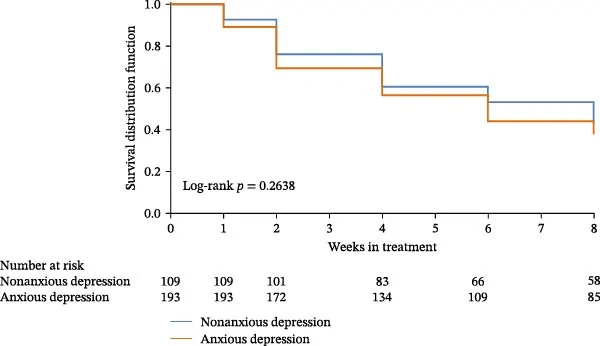
Time to response, by anxious versus nonanxious depression. Log‐rank *p* = 0.2638.

## 4. Discussion

Our study compared treatment outcomes for patients with anxious versus nonanxious depression. Both groups received similar doses of escitalopram or paroxetine, with no significant differences in treatment duration, indicating that these factors did not confound the results. Our findings did not support previous research suggesting that anxiety worsens depression treatment outcomes [4, 8–10]. In our study, we found no statistically significant differences in remission, response, or time‐to‐event outcomes between the two groups after 8 weeks. This outcome suggests that while anxious depression may manifest differently in terms of symptom severity, we did not find evidence that it significantly affects the magnitude of the treatment response. Similar results were reported in a study where anxiety symptoms, though linked to higher initial symptom severity, did not hinder treatment outcomes for anxious depression in the short or long term. That study employed a multidisciplinary treatment approach, including psychopharmacological medication and clinical management [21]. In contrast, our study used a single‐blinded design across multiple centers, further supporting that pharmacological treatment for anxious depression is as effective as for nonanxious depression. Moreover, a Korean study also supports our results, finding no worse outcomes for anxious depression in antidepressant treatment. However, this Korean study excluded patients with comorbid anxiety disorders, who showed less improvement on both the HAM‐D and HAM‐A scores. They also allowed concomitant medications [22]. In contrast, our study included patients with comorbid anxiety disorders and prohibited other psychotropic medications. These methodological differences strengthen the evidence that escitalopram and paroxetine provide additional cross‐cultural evidence for treating both anxious and nonanxious depression.

Although this historical cohort may not fully reflect contemporary Taiwanese clinical practice, it retains scientific value because of its multicenter design, standardized SCID‐based diagnostic assessment, and longitudinal clinician‐rated and self‐reported treatment measures. To our knowledge, it represents one of the few multicenter Taiwanese datasets examining both the clinical presentation and antidepressant treatment outcomes of anxious depression. Given the limited evidence from Asian outpatient populations, these data may contribute to a broader cross‐cultural understanding of anxious depression. Nevertheless, current diagnostic practice is based on DSM‐5/DSM‐5‐TR frameworks, and our HAM‐D‐based definition of anxious depression is not identical to the DSM‐5 anxious distress specifier. Accordingly, the present findings should be interpreted as complementary historical evidence rather than a direct representation of current epidemiology or clinical practice; prospective validation using contemporary diagnostic frameworks is warranted.

### 4.1. Limitations

Several limitations should be considered. First, changes in the sociocultural context, clinical practice, and treatment strategies over time may limit the generalizability of our findings to contemporary settings. Second, restricting the analysis to participants with sufficient follow‐up data through at least Week 4 and available outcome data may have introduced attrition or selection bias. Third, our study sample was limited to psychiatric outpatients, which may restrict the generalizability of the results to inpatient populations or those receiving care in community or psychiatric clinics. Fourth, anxious depression was defined using a psychometric cutoff, which may not fully capture its dimensional or diagnostic complexity. In addition, baseline sociodemographic and clinical differences between the anxious and nonanxious depression groups may have resulted in residual confounding, and the possibility of a Type II error cannot be excluded. Lastly, both groups received paroxetine and escitalopram, which are FDA‐approved treatments for anxiety and depression, meaning that the results may only apply to these specific medications and may not generalize to other antidepressants.

## 5. Conclusion

In conclusion, within this Taiwanese multicenter outpatient cohort, anxious depression was not significantly associated with lower remission or response rates or with a longer time to these outcomes during 8 weeks of treatment with escitalopram or paroxetine. These findings contribute to the limited evidence on longitudinal antidepressant outcomes among Asian outpatients with anxious depression and complement our previous characterization of its clinical presentation in this cohort. Although the historical nature of the dataset and baseline differences between groups warrant cautious interpretation, the findings provide complementary cross‐cultural evidence on anxious depression. Further research may help clarify the underlying mechanisms that govern the interaction between anxiety and depression in treatment contexts.

## Funding

This study was supported by Grant PH‐111‐PP‐02 from the National Health Research Institutes, a nonprofit foundation dedicated to medical research and improved healthcare in Taiwan, and Chang Gung Memorial Hospital Research Program (Grant CMRPG CORPG3L0021).

## Conflicts of Interest

The authors declare no conflicts of interest.

## Data Availability

The datasets generated or analyzed during the study are available from the corresponding author upon reasonable request.
